# Viromic Koch’s postulates resolve iron prawn syndrome etiology

**DOI:** 10.1128/jvi.00696-26

**Published:** 2026-07-31

**Authors:** Guohao Wang, Chengyan Zhou, Qingqing Zhou, Weifeng Shi, Hans Nauwynck, Xiaomeng Guo, Kathy F. J. Tang, Jie Huang, Xuan Dong

**Affiliations:** 1 State Key Laboratory of Mariculture Biobreeding and Sustainable Goods, Laboratory for Marine Fisheries Science and Food Production Processes, Key Laboratory of Maricultural Organism Disease Control, Ministry of Agriculture and Rural Affairs, Qingdao Key Laboratory of Mariculture Epidemiology and Biosecurity, Yellow Sea Fisheries Research Institute, Chinese Academy of Fishery Sciences, Qingdao Marine Science and Technology Center 117919, Qingdao, China; 2 College of Fisheries and Life Science, Shanghai Ocean University 74595, Shanghai, China; 3 Shanghai Institute of Virology, Shanghai Jiao Tong University School of Medicine 56694, Shanghai, China; 4 Ruijin Hospital, Shanghai Jiao Tong University School of Medicine 66281, Shanghai, China; 5 Laboratory of Virology, Faculty of Veterinary Medicine, Ghent University 26656, Ghent, Belgium; Wageningen University & Research, Wageningen, the Netherlands

**Keywords:** virome, Koch’s postulates, etiology, infectious precocity virus

## Abstract

Koch’s postulates remain unachievable for most crustacean viruses due to uncultivable pathogens and chronic infection dynamics. We propose viromic Koch’s postulates, a culture-independent framework, to address this limitation. The modified postulates uncover comprehensive information about viruses throughout the chain from clinical samples to viral preparations to challenge trials by combining the virome from metatranscriptomic, proteomic, or molecular analyses with diagnoses from clinical or pathological observations. Applying this approach to iron prawn syndrome (IPS), a chronic disease that causes stunted growth in *Macrobrachium rosenbergii*, we identified infectious precocity virus (IPV) as the exclusive viral correlate through metatranscriptomic analysis. IPV showed 100% prevalence in symptomatic prawns, but was absent in controls. Viral preparations from diseased tissue reproduced IPS pathology in healthy prawns upon inoculation, with IPV replication kinetics correlating to growth retardation. Histopathology and *in situ* hybridization localized IPV to neuroendocrine tissues, confirming tropism and replication sites. Our findings definitively establish IPV as the causative agent of IPS. Furthermore, this study demonstrates how systematizing existing culture-independent verification steps—referred to here as viromic Koch’s postulates—can serve as a practical, standardized operational roadmap for pathogen verification in uncultivable systems, facilitating the investigation of viral diseases in aquaculture.

**IMPORTANCE:**

Koch’s postulates remain difficult to apply to most crustacean and other uncultivable viruses because pathogen isolation and cell culture are often not feasible. To address this limitation, we propose “viromic Koch’s postulates,” a culture-independent framework that integrates whole-virome background exclusion, comprehensive inoculum characterization, and *in vivo* spatiotemporal validation of viral replication. Using this approach, we establish IPV as the etiological agent of IPS in *Macrobrachium rosenbergii*. By linking sequence-based discovery to biological causation without relying on virus culture, this work establishes an operational roadmap for rigorous pathogen identification in crustaceans and other host systems where traditional isolation-based criteria are not feasible.

## INTRODUCTION

Koch’s postulates, formulated by Robert Koch in the late 19th century, include a set of criteria designed to establish a causal relationship between a specific microorganism and a disease (1). The four postulates have laid the foundation for identifying pathogens responsible for many infectious diseases and have been proven instrumental in the study of bacterial illnesses (2). However, their limitations pose significant challenges for virology (3–5). Consequently, Koch’s postulates have been continuously refined to accommodate broader insights into host-microorganism relationships, including molecular Koch’s postulates (6), ecological Koch’s postulates (7), microecological Koch’s postulates (8), commensal Koch’s postulates (9), and metagenomic Koch’s postulates (10).

It is challenging to propagate and purify viruses without susceptible cell lines. This is especially critical in crustacean virology, where the lack of established cell lines fundamentally precludes true biological propagation and purification of viral pathogens. However, conventional purification methods (e.g., density gradient centrifugation, filtration) only physically separate virus particles. They cannot distinguish between viruses or viral strains with similar physical properties. Without cell culture-based biological purification techniques, such as plaque purification or endpoint dilution cloning, which segregate single, infectious strains using viral replication, obtaining a pure isolate of a specific replication-competent virus is extremely challenging. Consequently, it is unattainable for most crustacean viruses to fulfill Koch’s core postulates. Difficulties reproducing disease due to the need for environmental, host, and microbial (co-infections) factors impede definitive viral pathogen identification following Koch’s postulates (3).

It is noteworthy that the rapidly advancing technologies of metagenomics and metatranscriptomics have significantly accelerated the pace and scale of virus discovery (11–17). These approaches provide more comprehensive insights into the diversity and abundance of viruses, thereby serving as powerful tools for pathogen identification and characterization. However, despite the proposal of metagenomic Koch's postulates, there is a lack of systematic cases where strict experimental methods combined with high-throughput sequencing data have been used to definitively identify viral pathogens.

Iron prawn syndrome (IPS), a chronic disease in *Macrobrachium rosenbergii* characterized by sexual precocity and growth retardation, exemplifies the challenge to fulfill Koch’s postulates: the lack of susceptible cell lines preventing biological purification and the complexities of chronic infection with potential co-infections (18–20). Since its emergence in 2010, IPS has caused substantial economic losses in giant freshwater prawn aquaculture (21). Unlike mass mortality events in aquatic animals, IPS remains asymptomatic during the early stages of prawn cultivation, only manifesting clinical signs after reaching specific growth phases (21). Despite numerous etiological efforts for years, the pathogen of IPS remained elusive to date. We used histopathological analysis and infection experiments (using 0.22 μm membrane-filtered viral filtrate for immersion challenge) to demonstrate that IPS is likely caused by a viral pathogen (22). In addition, we identified a novel virus associated with IPS by metatranscriptomics, namely the infectious precocity virus (IPV) (22). IPV possesses a 12.6 kb ssRNA (+) genome, and phylogenetic analysis revealed that it represents a novel member between the Jingmenvirus group and *Flavivirus* (22). Our previous studies established a strong association between IPV and IPS through histopathology, challenge experiments, and molecular detection. However, because pure viral cultures could not be obtained, a more stringent culture-independent verification strategy was needed to further strengthen causal inference.

To address these challenges, we analyzed the “virome,” the complete set of viruses within *M. rosenbergii* samples with and without IPS. We sought to overcome the limitations of traditional methods and offer new approaches for pathogen identification in crustaceans, which would eventually help refine Koch’s postulates.

## RESULTS

### Collective analysis of virome libraries related to IPS studies

**Fig 1:**
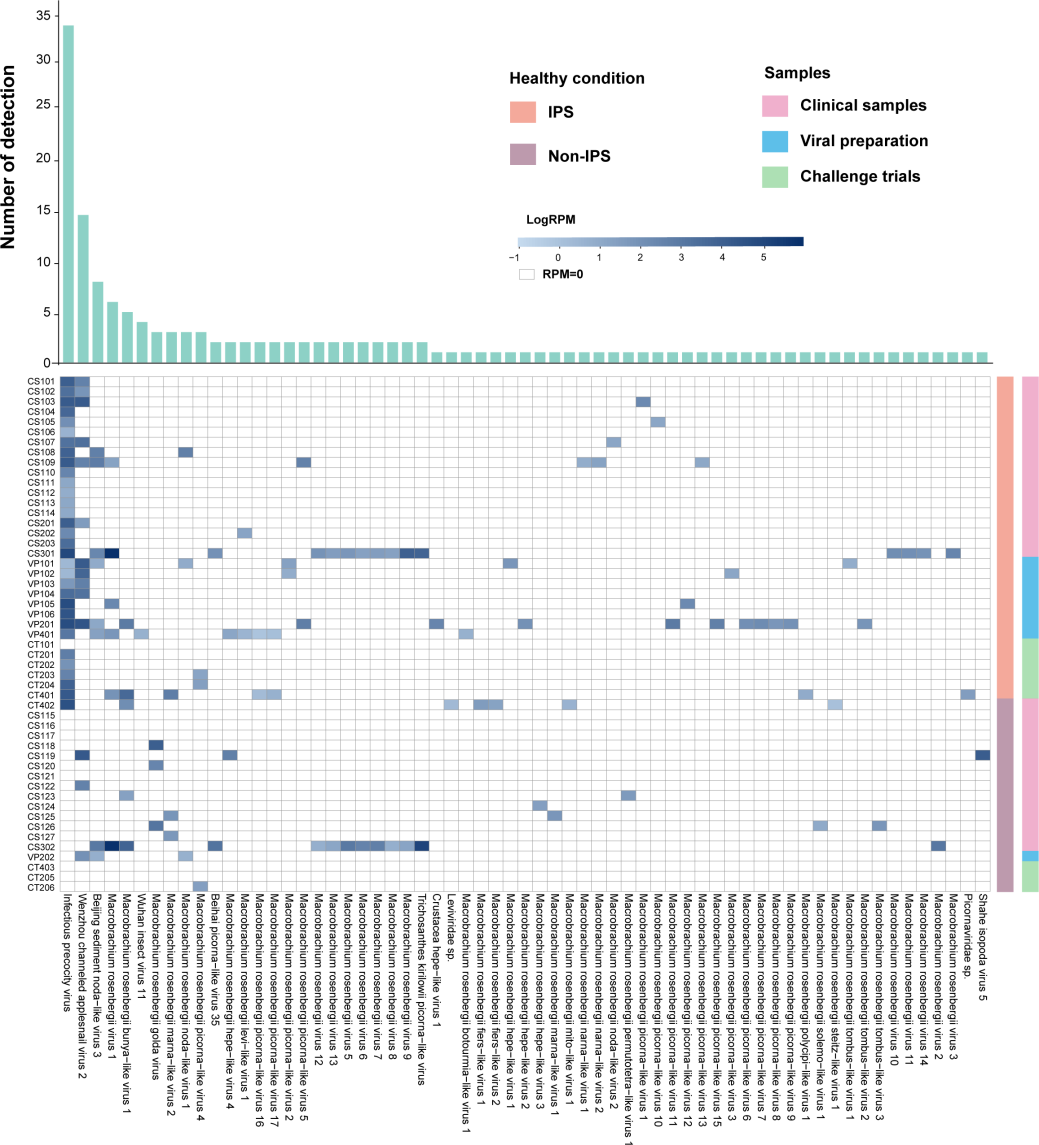
Virome characteristics of IPS-affected and non-IPS *M. rosenbergii* samples. The top bar chart represents the occurrence frequency of each RNA virus across all libraries. The heatmap below shows the absence (RPM = 0) or abundance (logarithmic RPM = 0–6) of each virus in individual libraries, with darker blue colors indicating higher abundance. Library names were listed on the left. On the right, stacked bars indicated the origin of the samples (clinical samples, viral preparations, and challenge trials) and their health conditions.

The case definition of IPS was defined in a previous study (22). With this definition, this study collected a total of 51 RNA-seq libraries between 2018 and 2025 in our published sequencing data (34 libraries), newly sequenced data in this study (4 libraries), and publicly available data sets uploaded by other researchers (13 libraries). These libraries from different sample sources of clinical samples, viral preparations (viral filtrate prepared from infected tissues), and challenge trials were categorized into 32 libraries from IPS-affected samples, 18 from non-IPS-affected samples, and a library of egg samples delivered from an IPS-affected spawner. Comprehensive virome profiling was conducted to identify and characterize all viruses present in each library. A total of 63 distinct viruses were identified across all metatranscriptomic libraries. Among them, the three most frequently identified viruses were IPV, Wenzhou channeled applesnail virus 2 (WCAV2), and Beijing sediment noda-like virus 3 (BSNV3) (Fig. 1). Apart from the three dominant viruses, the remaining 60 viruses exhibited substantially low identification rates: one virus was identified in six libraries, one virus was identified in five libraries, four viruses were identified in three libraries, 15 in two libraries, and 39 in only one library, respectively, with reads per million (RPMs) from 0.8 × 10^0^ to 8.8 × 10^5^ (Table 1). Notably, no virus was identified in the virome of the egg sample (20190123010-Luan); however, subsequent RT-qPCR detection of the spawner’s cephalothorax tissue of 20190123010, which showed IPS and delivered the egg sample, confirmed IPV’s presence at a viral load of 10^4.61 ± 0.07^ copies/μg-RNA.

**TABLE 1:** Detection of the top three viruses in different sample groups

Virus	Parameter	Clinical samples		Viral preparations		Challenge trials	
		IPS	Non-IPS	IPS	Non-IPS	IPS	Non-IPS
IPV	Detection rate	18/18	0/14	8/8	0/1	6/6	0/3
	RPM	4.7 × 10^0^–9.1 × 10^4^	0.0	2.8 × 10^0^–5.0 × 10^4^	0.0	8.0 × 10^1^–3.5 × 10^4^	0.0
WCAV2	Detection rate	6/18	2/14	6/8	1/1	0/6	0/3
	RPM	3.9 × 10^1^–1.5 × 10^4^	3.8 × 10^2^; 1.7 × 10^4^	4.9 × 10^2^–2.9 × 10^4^	9.0 × 10^1^	0.0	0.0
BSNV3	Detection rate	3/18	1/14	3/8	1/1	0/6	0/3
	RPM	2.4 × 10^2^– 6.9 × 10^2^	9.8 × 10^2^	8.5 × 10^0^–2.3 × 10^1^	6.1 × 10^0^	0.0	0.0

A principal component analysis (PCA) on the relationship between samples and the virome matrix in the above-mentioned 51 libraries showed that the samples were significantly clustered according to their health status (healthy vs. IPS), but not based on their library sources (Fig. S1), which indicates IPS-related health status is associated with viral infection, rather than sample sources.

### Clinical sample virome

IPV was consistently present in *M. rosenbergii* suffering from IPS. Prawns lacking IPV do not exhibit the corresponding clinical signs of IPS.

In 18 IPS-affected clinical samples from 14 farmed *M. rosenbergii* ponds collected in 3 provinces, IPV was identified in all of the samples, with RPMs ranging from 4.7 × 10^0^ to 9.1 × 10^4^. In contrast, 14 prawn samples collected from 5 farmed ponds in 2 provinces were found to be IPV free in their virome, and these samples did not show IPS clinical signs. However, other viruses identified in the virome showed inconsistency with IPS. For example, among 18 IPS-affected clinical samples, WCAV2 was identified in 33.3% (6/18), with RPMs ranging from 3.9 × 10^1^ to 1.5 × 10^4^. In the 14 non-IPS clinical samples, the identification rate of WCAV2 was 14.3% (2/14), with RPMs of 3.8 × 10^2^ and 1.7 × 10^4^. BSNV3 showed lower identification rates in both IPS and non-IPS groups. In 18 IPS-affected samples, the identification rate was 16.7% (3/18), with RPMs of 2.4 × 10^2^ to 6.9 × 10^2^. In the non-IPS samples, BSNV3 was identified from one sample (7.1%), with an RPM of 9.8 × 10^2^. Therefore, virome data confirmed IPV as the only virus consistently present in IPS-affected prawns. Based on the clinical sample virome, consequently, our investigation into the causative agent of IPS has been narrowed to focus on IPV.

### Viral preparation virome

Only IPV was consistently present in the viromes of the viral preparations from IPS-affected prawns but absent from the preparation from healthy prawns.

In total, nine viral preparations from eight IPS-affected prawn samples and one non-IPS sample were subjected to virome analysis by metatranscriptomic sequencing in this and our previous preparations (Fig. 1, sky blue marked). IPV was identified in all eight samples of viral preparations of IPS-affected prawns collected from four farms in two provinces, with RPMs ranging from 2.8 × 10^0^ to 5.0 × 10^4^; while WCAV2 was identified in 62.5% of the eight samples, with RPMs ranging from 4.9 × 10^2^ to 2.9 × 10^4^; and BSNV3 showed a lower identification rate of 37.5% (3/8), with RPMs of 8.5 × 10^0^ to 2.3 × 10^1^ (Table 1). In the viral preparation of a non-IPS sample, no IPV was present in the virome, but BSNV3 was identified with RPMs of 9.0 × 10^1^ and 6.1 × 10^0^ (Table 1).

**Fig 2:**
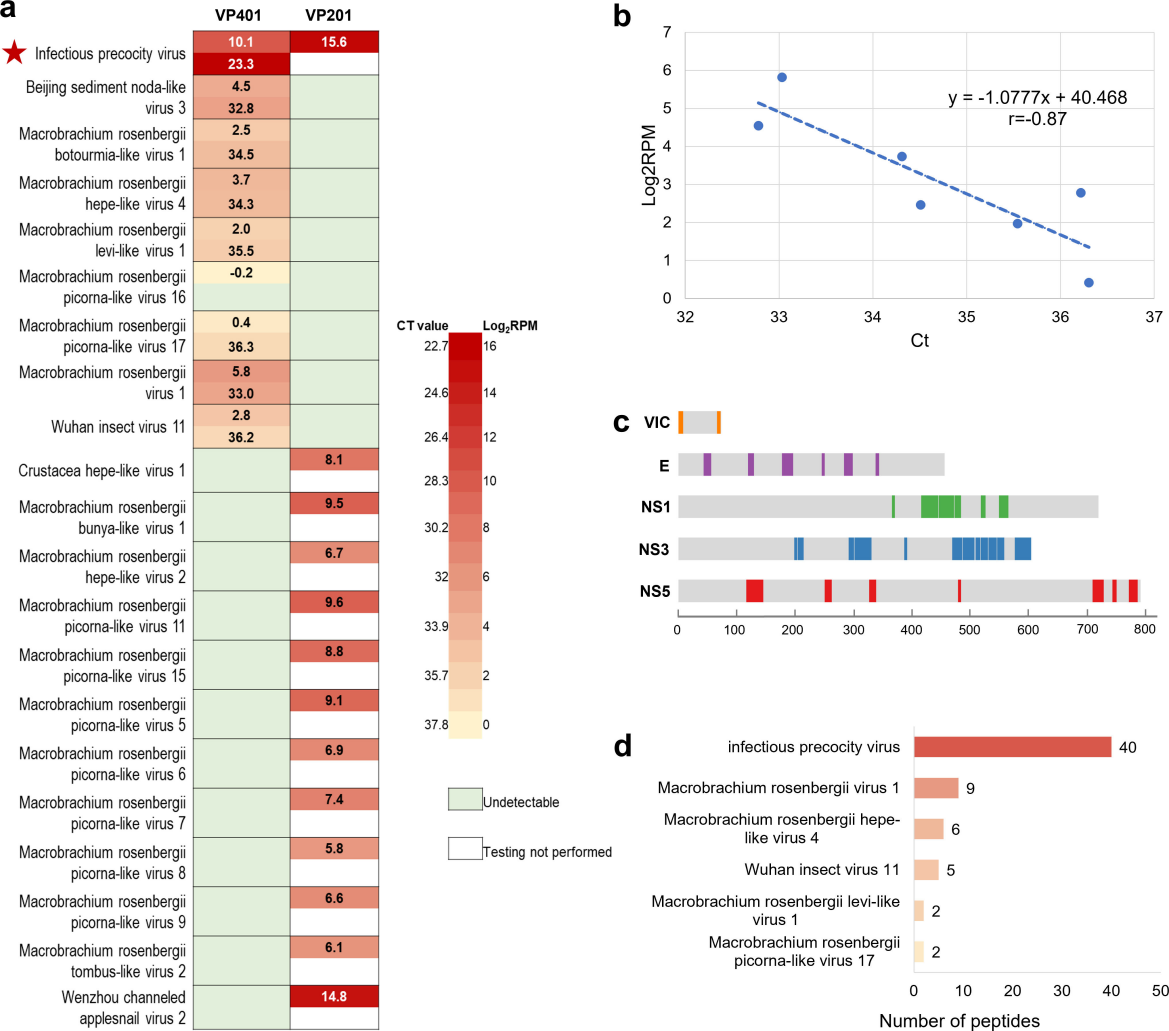
Comprehensive viromic characterization revealed by metatranscriptomics and proteomics of viral inoculum. (**a**) Comparison of the viral compositions of the inoculum used in the previous challenge experiment (VP201) and the IPV-dominant inoculum prepared in this study (VP401). In each cell, the upper number indicates viral abundance determined by virome (Log_2_RPM), and the lower number indicates the corresponding Ct value determined by SYBR Green qPCR. The color gradient shows the relationship between Ct value and Log_2_RPM; green indicates undetectable viruses, and white indicates that testing was not performed. (**b**) Correlation between viral abundance estimated by virome (Log_2_RPM) and SYBR Green qPCR Ct values in VP401. A significant negative correlation was observed (*r* = −0.87, *P* < 0.05), with the regression equation Log_2_RPM = −1.0777 × Ct + 40.468. (**c**) Proteomic validation of IPV structural and non-structural proteins via liquid chromatography-tandem mass spectrometry (LC-MS/MS). The primary sequence coverage maps indicate the specific amino acid positions of the identified peptides mapped to the IPV proteins: VirC, E, NS1, NS3, and NS5. (**d**) Quantitative comparison of the identified peptides across all identified viruses in the inoculum.

The metatranscriptomic data revealed the first inoculum (VP201) for the previous challenge experiment contained a complex mixture of 14 identified viruses, with IPV and WCAV2 in 60.9% and 35.0% dominances at RPMs of 5.0 × 10^4^ and 2.9 × 10^4^, respectively, while the other 12 viruses in a total of 4.2% relative abundance (Fig. 2a). Although inoculum VP201 successfully reproduced IPS in our previous study (22), the presence of multiple co-occurring viruses precluded a rigorous assessment of the specific etiological role of IPV. To address this limitation, in the present study, we deliberately selected sample CS110, whose virome only contained IPV, with an RPM of 3.2 × 10^2^, to use for the preparation of the inoculum to be used for the subsequent challenge trial. However, after the preparation operation, a total of nine viruses were identified in the virome (library: VP401), with an overwhelmingly dominant 1.1 × 10^3^ RPM of IPV, accounting for approximately 90.6% of the total viral RPMs. In stark contrast, the abundance of the remaining eight background viruses was negligible, with a total relative abundance of 9.5%, and RPM values ranging from merely 0.8 × 10^0^ to 5.6 × 10^1^ (Fig. 2a). Subsequent validation via SYBR Green qPCR confirmed the presence of seven background viruses (excluding Macrobrachium rosenbergii picorna-like virus 16), with Ct values spanning from 32.8 to 36.3. A robust linear relationship was identified between the Log_2_RPM values and qPCR Ct values (*r* = −0.87; *P* < 0.05; Log_2_RPM = (−1.077 ± 0.277) × Ct + (40.468 ± 9.603), the parameters are expressed as mean ± standard error) (Fig. 2b), demonstrating that the RPM values in virome sequencing data accurately reflected the amount of all viral copies in the samples. Notably, comparison of the two preparations also revealed that IPV was the only virus consistently shared between them (Fig. 2a). Therefore, this IPV-dominant preparation was selected as the inoculum for subsequent challenge experiments.

To exclude non-viral pathogens, the inoculum was filtered through a 0.22 μm membrane, and 50 μL of the filtrate was cultured on tryptic soy agar (TSA) and rose bengal agar at 28°C, yielding no colony growth (Fig. S2).

To verify whether these genomic data translate into the presence of virions and replication complexes of the focused virus, we used LC-MS/MS to identify virus-specific proteins in the above-selected inoculum used for the subsequent challenge experiment. For peptides, the screening criteria were set as peptide spectrum match (PSM) false discovery rate (FDR) ≤ 0.01. The results showed that a total of 40 peptides were identified and derived from structural and non-structural proteins of IPV, including VirC (3), M (1), E (6), NS1 (6), NS2A (1), NS3 (13), NS4A (1), NS4B (1), and NS5 (8). Adhering to the principle that a protein’s identification is considered reliable when detected by a minimum of two peptides, which is commonly referred to as the “two-peptide rule” (23), our findings confirmed the existence of protein products derived from the VirC, E, NS1, NS3, and NS5 proteins of IPV (Fig. 2c). Furthermore, to explicitly address the concern regarding potential false-positive protein identifications from host tissue carryover, we confirm that all reported viral peptide sequences were blasted against the *M. rosenbergii* reference proteome. None of the IPV-derived peptides identified in the inoculum showed homology to known crustacean proteins. Consistent with the viromic abundance data, peptides from other background viruses were detected only at trace levels, including Macrobrachium rosenbergii virus 1 (nine peptides), Macrobrachium rosenbergii hepe-like virus 4 (6 peptides), Wuhan insect virus 11 (5 peptides), Macrobrachium rosenbergii fiers-like virus 1 (2 peptides), and Macrobrachium rosenbergii picorna-like virus 17 (2 peptides) (Fig. 2d).

### Challenge trial virome

Inoculation of healthy prawns with the selected inoculum resulted in IPS, with IPV replication demonstrated in the virome of challenged prawns, while inoculation with the control inoculum free of IPV did not.

**Fig 3:**
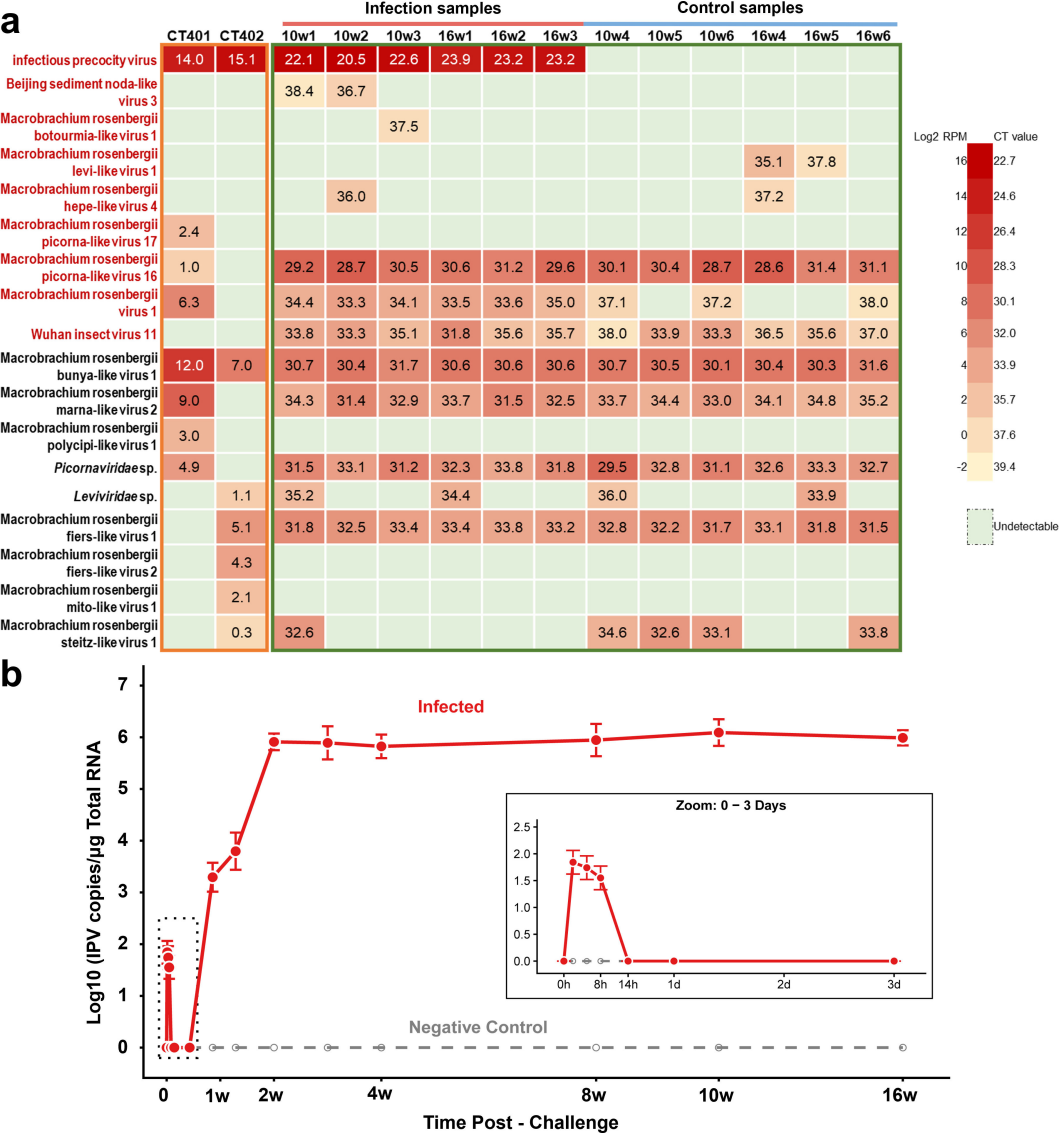
Virome profiling and *in vivo* replication kinetics identify IPV as the only consistently replicating virus associated with IPS. (**a**) Heatmap comparing viral profiles across groups. The orange box represents virome sequencing results, showing the Log_2_RPM abundance of IPV which establishes absolute dominance from 10 wpc (CT401) to 16 wpc (CT402). The green box displays qPCR Ct values from the current infection experiment. IPV exhibited massive, specific accumulation in all infected groups without substantial interference from background inoculum viruses, whereas the control group remained IPV-free. (**b**) *In vivo* replication kinetics of IPV. The line graph illustrates the IPV copies over 16 weeks. Data are presented as mean ± SD.

To evaluate the specific role of IPV in IPS, this study collectively analyzed the virome data from both our previous and present challenge experiments. In the previous challenge experiment, IPV maintained a dominant relative abundance ranging from ~95% to nearly 100%, with RPMs from 8.0 × 10^1^ to 7.8 × 10^3^, in the virome of the inoculum VP201-challenged individuals. Only minor background viruses, including *Macrobrachium rosenbergii* picorna-like virus 4, were identified at a relative abundance of 0.3% to 4.5%. The virome profiles from the present study were consistent with these previous findings. No viruses were identified in the virome from the control group sample (library: CT403). The metatranscriptomic data revealed that IPV became the dominant taxon (77.9%), with an RPM of 1.7 × 10^4^, in the virome of a challenged individual (library: CT401) after 10 weeks post-challenge (wpc), despite a transient emergence of Macrobrachium rosenbergii bunya-like virus 1 (19.1%), with an RPM of 4.1 × 10^3^. By 16 wpc (library: CT402), IPV established absolute dominance, with its relative abundance expanding to 99.5%, with an RPM of 3.5 × 10^4^ (Fig. 3a). To assess viral proliferation *in vivo*, we compared the Log_2_RPM values and qPCR Ct values of 12 samples from an infection experiment (six infected and six controls) based on the relationship established between Log_2_RPM and qPCR Ct values in the last section. The results demonstrated a massive and specific accumulation of IPV in all infected samples (Ct values ranging from 20.5 to 23.9, with an equivalent Log_2_RPM values ranging from 14.0 to 18.4), coupled with a complete absence of IPV in the control group (Fig. 3b). Meanwhile, the remaining background viruses from the inoculum, such as *Macrobrachium rosenbergii* virus 1 and Wuhan insect virus 11, were either restricted to extremely low levels or only sporadically detected in both groups. This confirms that the infection model was predominantly driven by IPV, without substantial interference from the trace background viruses. Besides, we monitored IPV loads at various time points with TaqMan qPCR to reveal the *in vivo* replication kinetics within the host. Following an initial detection, viral loads rapidly declined below the detection limit between 14 h post-challenge (hpc) and 1-day post-challenge (dpc), corresponding to a viral eclipse phase. Subsequently, a rapid exponential increase in viral load initiated at 3 dpc, ultimately stabilizing at a plateau of approximately 1.0 × 10^6^ copies/μg-RNA (Ct = 22.6 ± 1.08) from 2 wpc onward (Fig. 3b). The results of challenge trials indicated that inoculation with IPV-containing preparations can induce IPS. Both metatranscriptomic and qPCR analysis revealed IPV was the only consistent virus with significant replication in viromic data from previous and present challenge experiments, and the virome in the control groups did not contain it.

**Fig 4:**
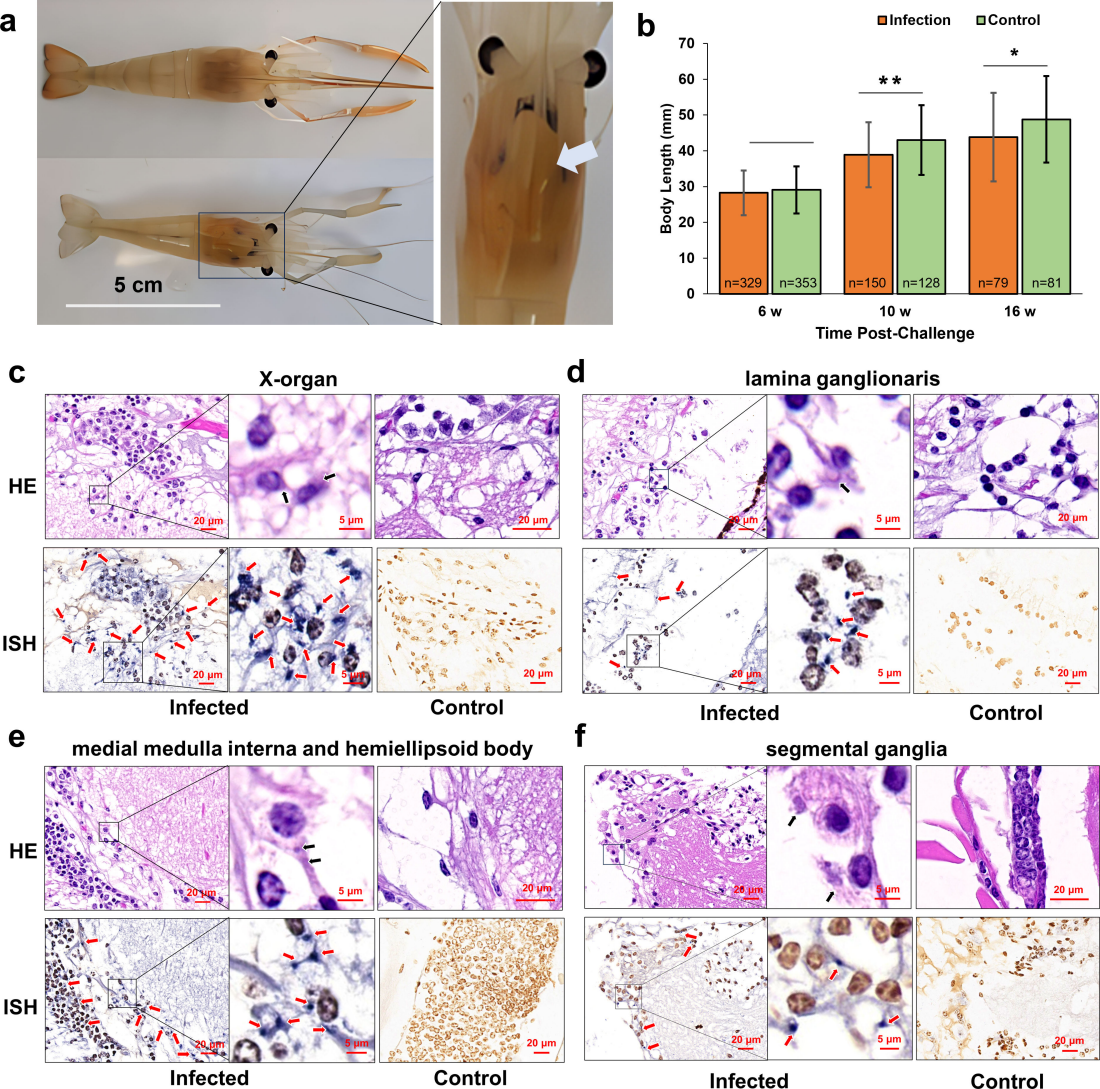
Recurrence of IPS by infection experiments. (**a**) Gross signs of female *M. rosenbergii* from challenge experiment in this study; above: a prawn in the control group, below: a prawn in the infected group, bar = 5 cm. (**b**) Statistical analysis of the body length of the prawns in the infected and control groups. *, *P* < 0.05; **, *P* < 0.001. (**c–f**) Cells in the X-organ, lamina ganglionaris, medial medulla interna and hemiellipsoid body of the compound eyes, and cells in the segmental ganglia of the cephalothoracic. Black arrows indicated cytoplasmic inclusions; red arrows indicated the hybridization signals. The middle image is the enlarged micrograph of the black box area in the left image. Scale bars  =  20 µm and 5 μm for low and high magnifications, respectively.

Consistent with the previous challenge trial (22), the present challenge trial also successfully reproduced the gross signs of IPS (Fig. 4a; Fig. S3). Compared to the control group without IPV, the infected male prawns exhibited shorter body lengths, and their claws appeared bluer and longer relative to their body size (Fig. S3). Meanwhile, the infected females exhibited earlier ovary maturation, with mature ovaries appearing at 12 wpc, whereas no mature ovaries were found in the control group at this stage (Fig. 4a). Body length measurements were recorded at 6, 10, and 16 wpc. At 6 wpc, mean body lengths were 29.08 mm (control group) and 28.26 mm (infected group), with no significant difference between the two groups. By 10 wpc, mean body lengths measured 43.03 mm (control group) and 38.89 mm (infected group), which was significantly shorter in the infected group compared to controls (*P* < 0.001). This significant difference persisted at 16 wpc, with mean lengths of 48.79 mm (control group) and 43.84 mm (infected group) (*P* < 0.05), respectively (Fig. 4b).

Through histopathological examination, eosinophilic inclusion bodies (indicated by the black arrow) were observed in the cytoplasm of neuroendocrine cells and globuli cells near the X-organ (Fig. 4c), cells in the lamina ganglionaris (Fig. 4d), globuli cells near the periphery of the medial medulla interna and hemiellipsoid body of the compound eyes (Fig. 4e), and cells in the segmental ganglia of the cephalothorax (Fig. 4f) of *M. rosenbergii* in the infected group. No similar eosinophilic particles were found in the control group. Due to the dispersed anatomical nature of shrimp neuroendocrine tissues, precise quantification of inclusion-bearing cells is technically constrained. Therefore, histological observations in this framework serve primarily as qualitative indicators of pathognomonic sign reproduction, while the definitive nature of these viral replication sites is strictly validated at the molecular level by targeted *in situ* hybridization (ISH). The results showed that blue-purple hybridization signals (indicated by the red arrow) with the IPV digoxigenin (DIG)-labeled RNA probe appeared in the cells near the X-organ (Fig. 4c), medial medulla interna and hemiellipsoid body (Fig. 4d), lamina ganglionaris (Fig. 4e), and segmental ganglia (Fig. 4f), which was consistent with the histopathological features. No signals were detected in the controls.

## DISCUSSION

The definitive assignment of a viral etiology to a disease has long relied on Koch’s postulates, a framework fundamentally constrained by the requirement for pathogen isolation and cultivation. For the vast majority of viruses, particularly those infecting crustaceans and other organisms lacking susceptible cell lines, this has rendered causal inference circumstantial. Our initial identification of IPV in *M. rosenbergii* affected by IPS established a strong epidemiological and molecular association but fell short of definitive proof for this very reason (22, 24). Recent studies have accelerated culture-independent host-pathogen identification through metagenomics and metatranscriptomics, particularly in systems where cultivation or purification is difficult. These approaches enable direct-from-sample pathogen detection and genomic characterization (15, 25). However, they also underscore a persistent challenge: identifying possible pathogens is increasingly feasible, whereas establishing rigorous causality remains substantially more difficult.

### Viromic Koch’s postulates

**Fig 5:**
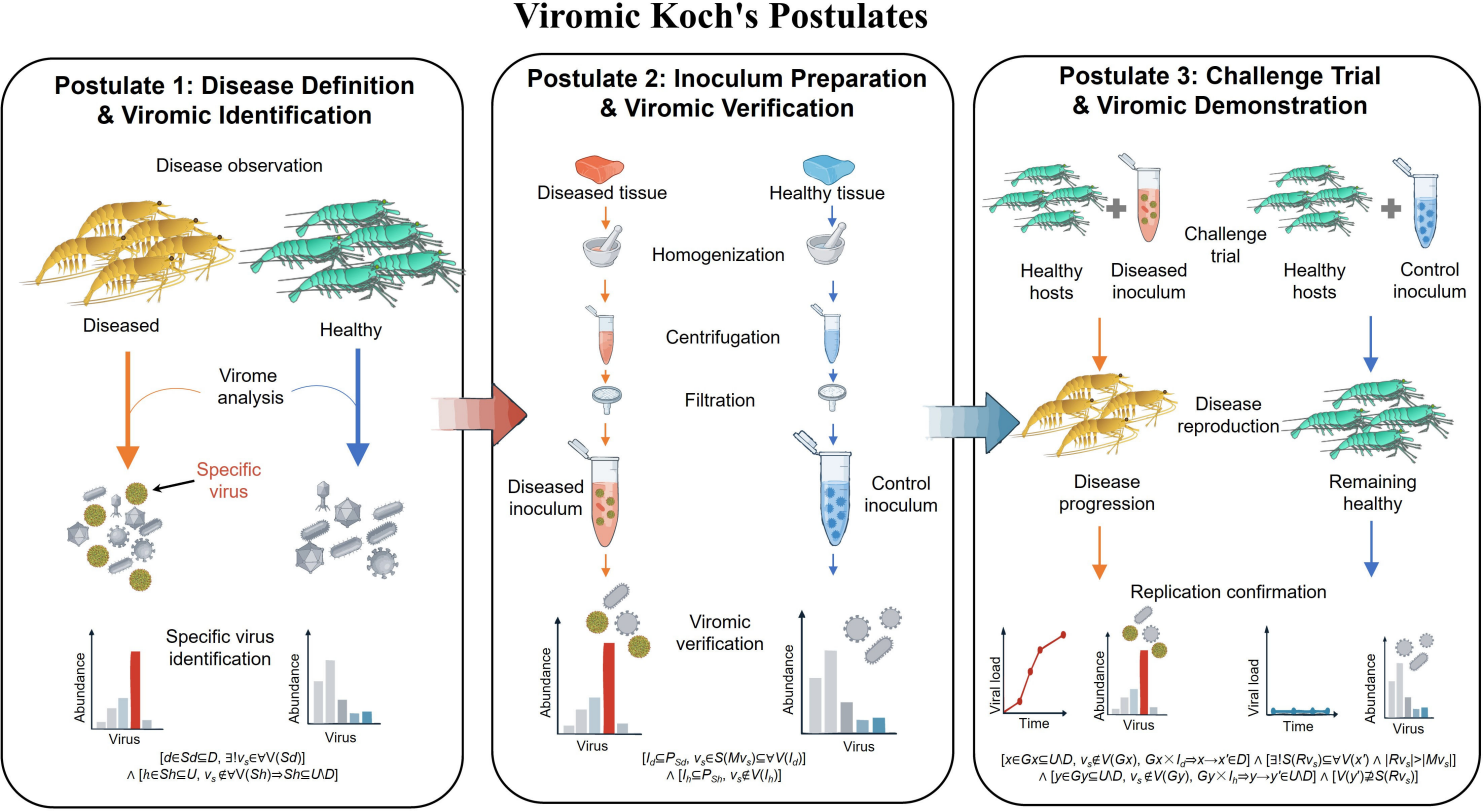
Schematic diagram of viromic Koch’s postulates. (Postulate 1) A specific virus is consistently present in the virome of individuals suffering from a particular disease. Conversely, individuals lacking this virus in their virome do not exhibit the corresponding clinical signs of the disease. (Postulate 2) Only the specific virus was consistently present in the virome(s) of the viral preparation(s) from individuals suffering from the disease but absent in that from individuals where the specific virus was not detected. (Postulate 3) Challenge of specific virus-negative individuals with the inoculum containing the specific virus must result in the disease, while control with the inoculum lacking the specific virus must not; together with viromic demonstration of the specific virus replication only in challenged individuals.

To overcome the pervasive limitations of traditional Koch’s postulates, we combine comprehensive virome data and validate a specific virus as the only one passing the postulates to bypass the cultivation-dependent feature of the original Koch’s postulates. This methodology is formalized into a cultivation-independent framework, termed “viromic Koch’s postulates” (Fig. 5), based on the present systematic process used to establish IPV as the definitive causative agent of IPS. This framework integrates information uncovered by viromic analyses in clinical samples, viral inocula, and challenged trials to validate that only one focused virus can pass three key postulates to identify the viral pathogenic agent of a defined disease of organisms, for which *in vitro* cell culture is not feasible to produce a biologically pure viral isolate through plaque or limiting dilution. The viromic Koch’s postulates are defined as follows.

#### Postulate 1

Postulate 1 states that a specific virus is consistently present in the virome of individuals suffering from a particular disease; conversely, individuals lacking this virus in their virome do not exhibit the corresponding clinical signs of the disease.

This postulate can be expressed in a set-theoretical formula:


$$P1=[d\in Sd\subseteq D,\exists !v_{s}\in \forall V(Sd)]\wedge [h\in Sh\subseteq U,v_{s}\notin \forall V(Sh)\Rightarrow Sh\in U\setminus D]$$

where *V* is the set of all viruses in the virome, *v*_s_ is the specific virus, *U* is the universal set of all individuals, *D* is the set of individuals showing clinical signs of the disease, *U*\*D* is the difference set from *U* without *D*, *Sd* or *Sh* is the set of sampled individuals respectively showing or not showing the clinical signs of the disease, *V*(*d*) and *V*(*h*) are the sets of viruses in the virome of individuals *d* and *h*, ∃! means “there exists exactly one,” ∀ means “for all,” ∈ means “is an element of,” ∉ means “is not an element of,” ⊆ means “is a subset of,” ⇒ means “implies,” and ∧ means “and.”

This postulate is a modification of the first postulate of Koch’s original postulates, which required identification of the microorganism in all diseased individuals but not in healthy ones. The modified postulate utilizes viromic data to demonstrate that all symptomatic individuals share a common virus in their virome, which captures the interest of investigators. Meanwhile, individuals free from the specific virus do not exhibit clinical signs. Several issues arise concerning individuals affected by viral diseases: co-infections (19, 22, 26), overlapping clinical signs (27), and latent asymptomatic carriers (28, 29). This amendment addresses these issues by focusing on the specific virus exclusive to the diseased individuals, as determined from viromic data. To avoid confusion from similar clinical signs or mortality caused by different pathogens, the case definition of a disease needs to be carefully determined (30). Selection of diseased samples should follow the case definition based on clinical signs (Level I diagnosis) and, if possible, histopathology (Level II diagnosis) (31). In strictly defined typical cases, the specific virus should be strongly associated with the case group. It is important to note that “strongly associated with” is a conceptual requirement reflecting strong epidemiological association rather than a universal statistical threshold. The statistical threshold depends on factors such as causality, the specificity of the case definition, and co-infection effects; it may vary significantly across different diseases and require comprehensive adjustment based on the specific circumstances of each case. Rather than setting a universal statistical threshold, distinguishing a specific virus from occasional viruses relies on two key metrics: (i) a statistically significant difference in viral prevalence between diseased and healthy cohorts and (ii) viral load dynamics, where the specific virus exhibits exponentially higher abundance in symptomatic hosts. When multiple viruses co-occur, sampling across diverse epidemiological sources is crucial for isolating the true etiological agent. For instance, restricted sampling in our study (e.g., only samples CS109 and VP201) initially implicated multiple viruses including IPV, WCAV2, and a picorna-like virus. However, independent multi-source sampling successfully filtered out this localized noise, confirming IPV as the sole consistent specific virus. Future studies using co-infection experiments with varying viral doses will be needed to determine whether WCAV2 or other viruses can exacerbate IPV-induced pathology or influence disease phenotype.

#### Postulate 2

Postulate 2 states that only the specific virus was consistently present in the virome(s) of the viral preparation(s) from individuals suffering from the disease but absent in that from individuals where the specific virus was not detected.

This postulate can be expressed in a set-theoretical formula:


$$P2=[I_{d}\subseteq P_{Sd},v_{s}\in S(Mv_{s})\subseteq \forall V(I_{d})]\wedge [I_{h}\subseteq P_{Sh},v_{s}\notin V(I_{h})]$$

where *I_d_* and *I_h_* are the sets of inoculum(s) selected from the viral preparations from the individual(s) *d* and *h* as described in P1, *P_Sd_* and *P_Sh_* are the sets of all preparations from relevant sets of *Sd* and *Sh*, *S*(*Mv*_s_) is the support set of the multiset *Mv*_s_ of infectious virions of the specific virus, *V*(*I_d_*) and *V*(*I_h_*) are sets of viruses in the viromes of those inoculums after laboratory preparation (e.g., filtration, centrifugation).

This modified second postulate eliminates the traditional requirement for *in vitro* pure culture isolation. Instead, viromic profiling is utilized to verify that the specific virus is present in the diseased inoculum and strictly absent from the control inoculum prepared from the individual(s) where the specified virus was not detected. While absolute biological purification requires susceptible cell lines (32–34), physical separation methods must be employed to enrich viruses and eliminate bacteria, fungi, parasites, and toxins. These include size-selective filtration (e.g., using a 0.22 µm membrane or pore sizes tailored to the specific virus) and differential or density gradient centrifugation. A critical challenge during inoculum preparation is the viral “concentration effect.” Physical enrichment may inadvertently amplify trace specific viruses in seemingly healthy donors (24) or co-concentrate multiple non-target viruses in diseased samples. The diseased inoculum should ideally differ from the control inoculum only by the presence of the specific virus. If multiple specific viruses co-purify, researchers must either source samples from diverse epidemiological backgrounds to find single-infection cases, or apply advanced separation strategies (e.g., density gradient centrifugation combined with limiting dilution or size-based separation) to isolate the target pathogen’s effect.

#### Postulate 3

Postulate 3 states that challenge of specific virus-negative individuals with the inoculum containing the specific virus must result in the disease, while control with the inoculum lacking the specific virus must not, together with viromic demonstration of the specific virus replication only in challenged individuals.

This postulate can be expressed in a set-theoretical formula:


$$P3=[x\in Gx\subseteq U\setminus D,v_{s}\notin V(Gx),Gx\times I_{d}\Rightarrow x\to x\prime \in D]\wedge [\exists !S(Rv_{s})\subseteq \forall V(x\prime )\wedge |Rv_{s}|>|Mv_{s}|]\wedge [y\in Gy\subseteq U\setminus D,v_{s}\notin V(Gy),Gy\times I_{h}\Rightarrow y\to y\prime \in U\setminus D]\wedge [V(y\prime )⊉S(Rv_{s})]$$

where *Gx* or *Gy* means the set of individual groups used for the challenge trails, *V*(*Gx*) or *V*(*Gy*) means the set of viruses in the viromes from *Gx* or *Gy*, × means the set of individuals to be challenged with the set of inoculum, → means “transition into” or “becomes,” *x*′ ∈ *D* or *y*′∈ *U*\*D* is the individual *x* or *y* being induced by the experimental challenges to sets of *D* or *U*\*D*, *S*(*Rv*_s_) means the support set of the multiset *Rv*_s_ of the replicated virions of the specific virus, |*Rv_s_*| and |*Mv*_s_| mean the numbers of *v*_s_ in the relevant multisets, and ⊉ means the set does not contain the following set as a subset.

This postulate combines and modifies the third and fourth postulates of Koch’s original postulates. It further validates pathogenicity through experimental challenge and demonstrates infection with the specific virus in challenged individuals without requiring re-isolation of the virus in a susceptible cell line. The viral inoculum must induce specific clinical signs and histopathological features of the disease, while the specific virus-free inoculum cannot; there should also be evidence of active viral replication in diseased individuals, thereby fulfilling the criteria for establishing causality. If multiple specific viruses are present in the inoculum, the pathogenic role of a particular virus can be determined using specific infection-blocking experiments, such as treatment with antiviral drugs, neutralizing antibodies, or RNA interference (RNAi) targeting that virus (35).

Formalization of viromic Koch’s postulates in set-theoretical terms allows formal definitions of the relationship between the presence of a specific virus and the occurrence of a particular disease, thereby verifying the logical rigor of the postulates. The three postulates, when combined under these set-theoretic terms, are logically rigorous and consistent to verify that only the specific virus can pass the three postulates. They form a valid framework where postulate 1 defines the specific virus-symptom relationship, postulate 2 ensures experimental accessibility, and postulate 3 demonstrates causation. The asymptomatic carrier case is correctly accounted for, and no postulate contradicts another.

Viromic Koch’s postulates set a culture-independent approach by comprehensively uncovering transparent information of all viruses to identify the causative agent of a particular viral disease. It not only differs from the original postulates, but also is distinct from other newly modified postulates. For example, metagenomic postulates rely on *in silico* sequence correlations and still demand purification of novel traits. Moreover, sequence correlation does not equate to *in vivo* causation (10). Our framework validates true *in vivo* causation. Ecological postulates emphasize population-level patterns to establish causality at the individual host level through experimental infection (7). Furthermore, whereas molecular postulates require pathogen cultivation and genetic manipulation (6), our approach is specifically tailored for uncultivable viruses. Collectively, these distinctions highlight how the viromic Koch’s postulates expand and adapt traditional concepts to accommodate the complexities of uncultivable viruses and the insights provided by modern sequencing technologies.

Application of this framework to IPS firmly establishes IPV as the causative agent and provides a practical strategy for resolving similar etiological challenges in uncultivable viral systems. The identification of IPV as the definitive etiological agent of IPS is supported by a convergence of virome tracking in the chain of clinical samples, viral preparations, and challenge trials. Although crude inocula were used in our two independent challenge experiments, both successfully replicated IPS symptoms. Crucially, virome analysis revealed that IPV was the only virus passing all three postulates and shared among all disease-related virome libraries from clinical samples, two independent preparations, and challenge trials, while absent in all health virome libraries. By mutually validating these infection results, we effectively ruled out the pathogenic role of any other background viruses. Additionally, the distinct *in vivo* replication kinetics of the inoculated viruses provided further evidence for this conclusion. Longitudinal virome tracking after challenge demonstrated that the minor background viruses failed to persist and were gradually cleared by the host. In contrast, only IPV established persistent, high-level replication in the diseased shrimp. Finally, large-scale industrial data provide a practical reverse validation of causality. Following the widespread implementation of IPV-targeted biosecurity measures, the incidence of IPS across the Chinese *M. rosenbergii* industry has plummeted from approximately 50% in 2019 to 5% at present (unpublished data). Collectively, the combination of viromic, clinical, and pathological information from epidemiological and experimental samples involved in the whole viromic Koch’s postulates approach robustly confirms IPV as the etiological agent of IPS.

### Conclusions

In conclusion, by applying viromic Koch’s postulates, we have identified IPV as the etiological agent of IPS. The core methodological components of this framework, including whole-virome background exclusion, comprehensive inoculum characterization, and spatiotemporal validation of *in vivo* replication kinetics, are not inherently limited to a specific host system. More broadly, this study provides a generalizable and rigorous pathway for establishing viral causality in the vast majority of hosts where cell culture is unavailable. Beyond its immediate application in aquaculture disease management, this integrated framework bridges the critical gap between sequence-based viral discovery and biological causal verification. Ultimately, it serves as a standardized, practical operational roadmap for resolving complex etiologies in diverse uncultivable invertebrate and wildlife systems.

## MATERIALS AND METHODS

### RNA-seq library consolidation from IPS and non-IPS individuals

In this study, we consolidated RNA-seq libraries from various sources to investigate the virome characteristics of *M. rosenbergii* populations affected with and without IPS. A total of 51 libraries were analyzed, including 32 libraries from IPS-affected samples and 18 from non-IPS samples, and an egg sample delivered from an IPS-affected spawner (19, 20, 22). These libraries were derived from our previously sequenced raw metatranscriptomic data in published studies (19, 22), newly metatranscriptomic sequencing in this study, and publicly available raw data sets uploaded by other researchers (20). Detailed metadata for all the libraries, including sample origin, health status, and sequencing platforms, were provided in Table S1.

### Virome analysis

Virome analysis was performed on the libraries according to the previously published method (36). The raw sequencing reads were first processed to remove adapters and for quality control using Trimmomatic (37), which is integrated within the Trinity platform (38). The resulting high-quality reads were then assembled *de novo* using Trinity with its default parameters. All assembled contigs were searched against the non-redundant protein (nr) database from GenBank via BLASTx, applying an E-value cut-off of 1 × 10⁻⁵ (39). To minimize false-positive identifications, contigs initially flagged as potentially viral were further validated by comparison against comprehensive non-redundant nucleotide and protein databases (12, 40). Confirmed viral contigs were merged and extended into longer sequences using Bowtie and Geneious (https://www.geneious.com) (40–42). For all the libraries, we standardized the virome composition and RPM values using the same analytical pipelines to ensure comparability across data sets (36). To systematically exclude endogenous viral elements (EVEs), all identified viral contigs were mapped against the *M. rosenbergii* reference genome. Sequences exhibiting significant chromosomal integration were classified as EVEs and filtered out of the analysis. The RPM values were provided in Table S2. Furthermore, to verify the influence of different source samples on the analysis results, we used the limma R package to perform PCA on the virome matrix.

### Preparation of viral inoculum

The viral preparation was carried out as previously described (22). Briefly, about 30 g of cephalothoraxes was homogenized in 200 mL phosphate-buffered saline (PBS), centrifuged at 10,000 × *g* at 4°C for 30 min, and the supernatant was retained. After the precipitate was washed with 50 mL PBS, it was homogenized and centrifuged again; this was repeated twice. The supernatant was combined. The supernatant was subjected to sterile filtration through a 0.22 μm membrane filter and metatranscriptomic sequencing. The preparation of *M. rosenbergii* (library: CS110) with IPV demonstrated in the virome was selected as the viral inoculum for the subsequent challenge assay. To verify the removal effect of bacteria and fungi, 50 μL of viral inoculum was coated on TSA and Bengal red plates and cultured at 28°C for 1 day and 5 days, respectively.

### Mass spectrometry analysis of viral inoculum

The viral inoculum was mixed with SDS-PAGE sample loading buffer (Beyotime, China), boiled in boiling water for 10 min, and then centrifuged at 9,560 × *g* for 3 min. The supernatant was subjected to polyacrylamide gel electrophoresis, and the gel strips were sent to Shanghai Bioprofile Biotechnology Co., Ltd (Shanghai, China) for mass spectrometry analysis. The mass spectrometric data were processed using MaxQuant (version 1.6.0.16). The outcomes of the database search were subjected to stringent criteria to minimize errors, with the results filtered and exported at a false discovery rate of less than 1%, applicable to both the peptide-spectrum-matched and protein levels.

### Challenge experiments

Healthy *M. rosenbergii* (average body length of 0.6 cm) without IPV were collected from a farm in Jiangsu province, China. These prawns were tested to verify no major freshwater prawn and shrimp pathogens listed by the World Organization for Animal Health (WOAH). The challenge assay with the above-mentioned viral inoculum was carried out as previously described (22). Prawns in both the infection and control groups were maintained at 28°C ± 1°C. The cephalothorax tissues were dissected and preserved with RNAlater (TIANGEN, Beijing, China) for RT-qPCR detection and 4% paraformaldehyde (PFA) fixative for histopathology and *in situ* hybridization. Meanwhile, the body length of each prawn was measured using photographs taken in shallow water with a ruler.

### qPCR detection method of IPV and other identified viruses

The RT-qPCR method was established in previous studies (24). The assay employed specific primers and a probe targeting IPV, with the forward primer sequence being 5′-AGG AGA GGG TTT TGG CTT G-3′, the reverse primer sequence 5′-CTG GAT TGG AAG GGA ACT CTG-3′, and the probe labeled with FAM and TAMRA (5′-FAM-CCG CGA CAC TTA CAA CTG CCC TT-TAMRA-3′). The standard curve was generated with the equation Ct = −3.406 Log_10_(IPV copies/reaction) + 37.053, with an *R*² of 0.999, an amplification efficiency of 96.6%, and a limit of detection of one copy per reaction. To ensure RNA quality, the concentration and purity were assessed using a NanoDrop 2000 spectrophotometer (Thermo Scientific, USA). Besides, virus-specific SYBR Green qPCR assays were conducted to verify the presence of inoculum-introduced viruses identified by virome sequencing. For each sample, 500 ng of total RNA was first reversely transcribed into cDNA, which was subsequently used as the template for qPCR analysis. Primers targeting the ORF region of each viral sequence were designed using Geneious Prime, and the detailed primer sequences are listed in Table S3. The qPCR amplification program consisted of an initial denaturation step at 95°C for 2 min, followed by 40 cycles of 95°C for 10 s and 60°C for 30 s. Melt curve analysis was then performed following incubation at 95°C for 15 s and 60°C for 1 min, with the temperature gradually increased to 95°C at a ramp rate of 0.5°C/s. Amplification signals were evaluated according to the cycle threshold (Ct) values obtained for each assay, and samples yielding detectable Ct values were considered positive for the corresponding viral target.

### Histopathology and *in situ* hybridization

The cephalothoraxes collected in the challenge tests were immersed in 4% paraformaldehyde fixative for 24 h and then transferred to 70% ethanol. Tissue samples were processed histopathologically according to the criteria described by Lightner (43). ISH was performed as previously described (22).

### Statistical analysis

Statistical analyses were performed using Microsoft Excel 2021. An F-test was used to assess homogeneity of variances, followed by a two-tailed independent-samples t-test to compare prawn body length between the infected and control groups. Statistical significance was set at α = 0.05. Data are presented as mean ± SD.

## Data Availability

The raw data from the metatranscriptomic sequencing generated in this study are available in the NCBI Sequence Read Archive (SRA) database (Table S1).

## SUPPLEMENTAL MATERIAL

Supplemental figures jvi.00696-26-s0001.pdf Fig. S1 to S3.

Supplemental tables jvi.00696-26-s0002.xlsx Tables S1 to S3.

ASM does not own the copyrights to Supplemental Material that may be linked to, or accessed through, an article. The authors have granted ASM a non-exclusive, world-wide license to publish the Supplemental Material files. Please contact the corresponding author directly for reuse.
