# Supplementary material for: Viromic Koch’s postulates resolve iron prawn syndrome etiology
Source: J Virol. 2026 Jul 31;100(8):e00696-26. doi: 10.1128/jvi.00696-26 (PMC13483384; doi:10.1128/jvi.00696-26)
Supplement: Supplementary file 1 [file jvi.00696-26-s0001.pdf]

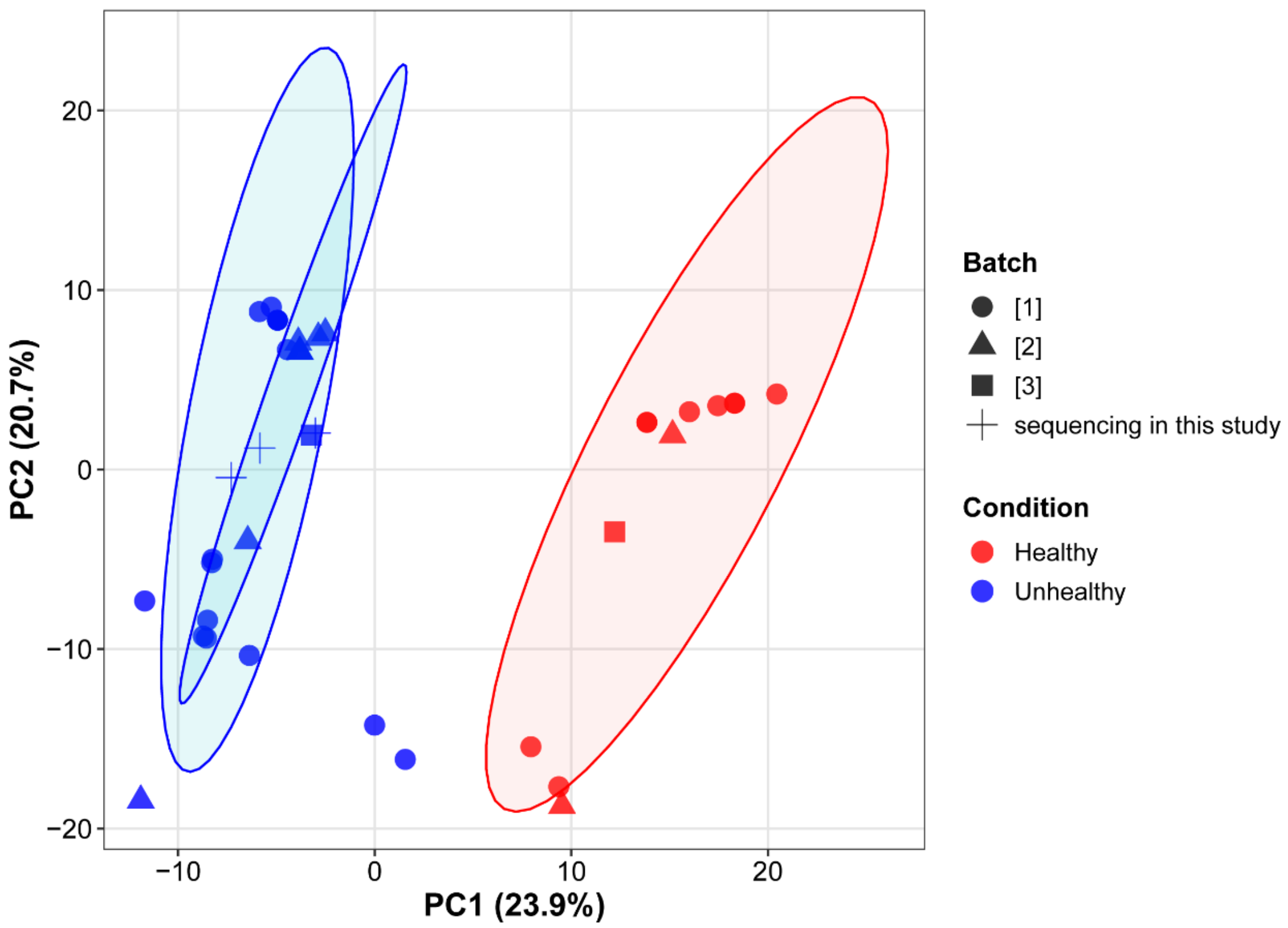

Fig S1 PCA of the batch-corrected virome matrix.

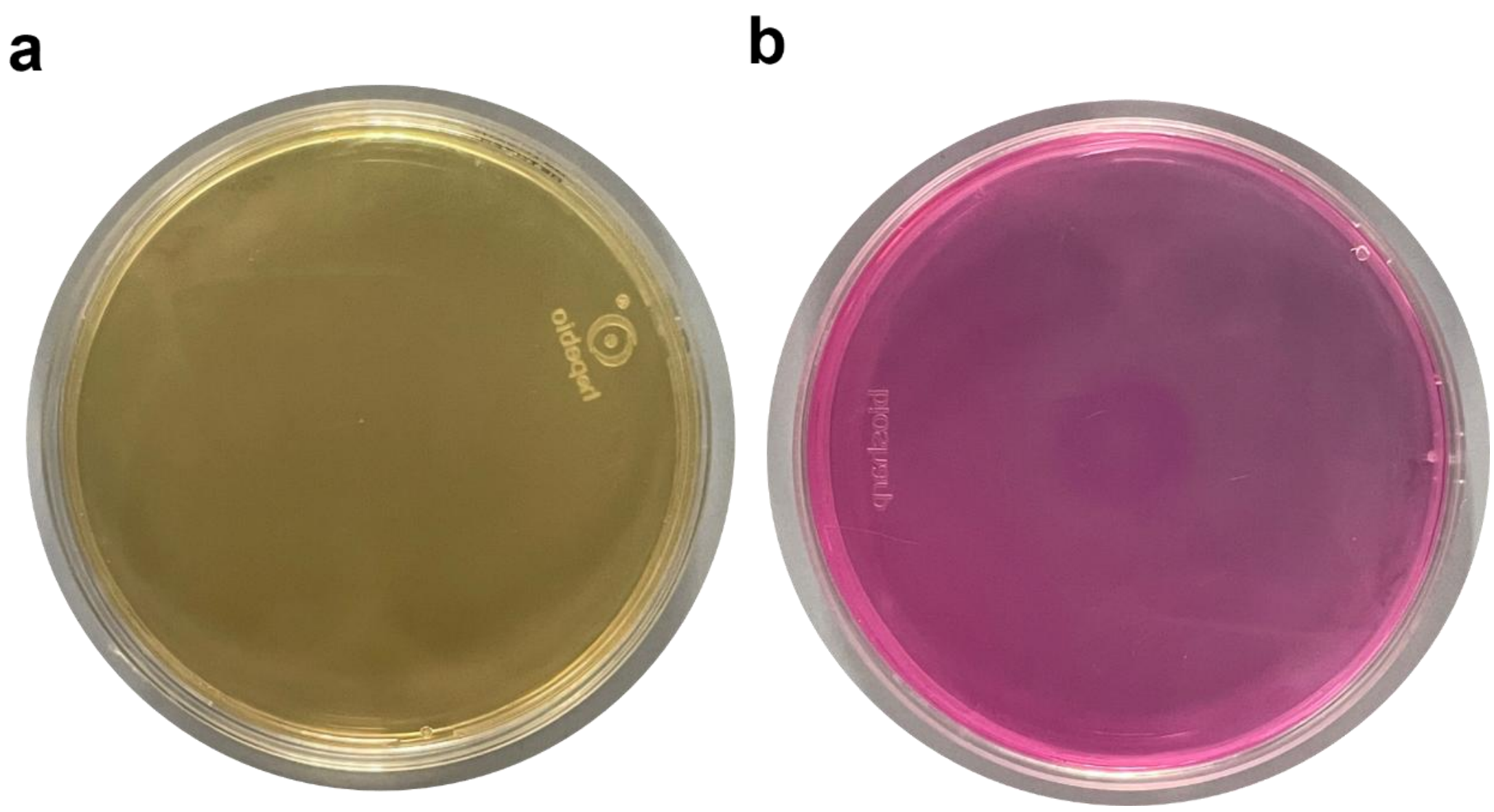

Fig S2 Validation of the inoculum sterility. The 0.22 μm-filtered inoculum was cultured on Tryptic Soy Agar (TSA) (a) and Rose Bengal Agar (b) at 28°C.

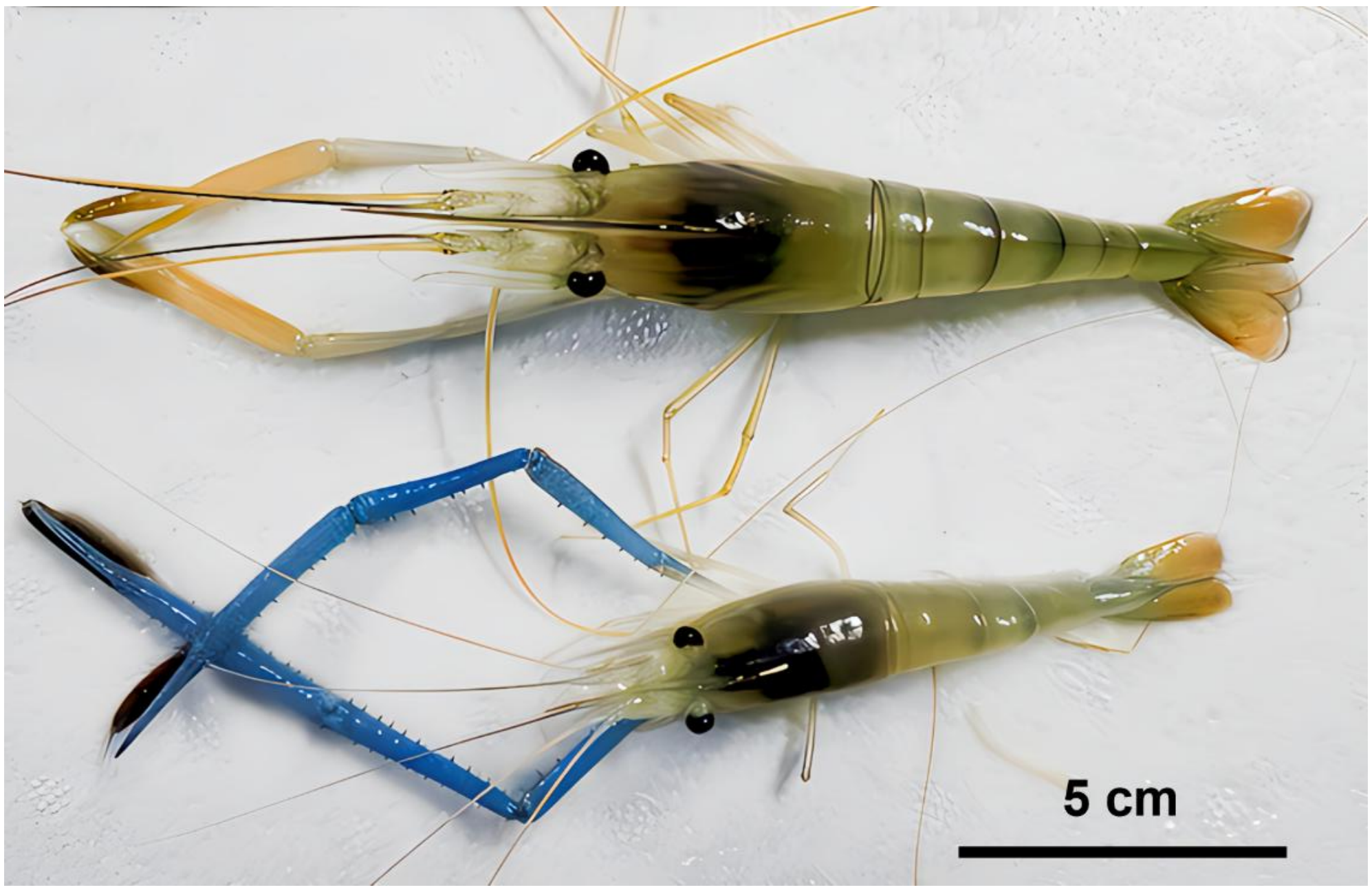

Fig S3 Gross signs of male *M. rosenbergii* challenged with the IPV preparation; top: prawn from the control group, below: prawn from the infected group.
